# Hyperprolactinemia after laparoscopic ovarian drilling: An unknown phenomenon

**DOI:** 10.1186/1477-7827-3-31

**Published:** 2005-08-07

**Authors:** Mohammad E Parsanezhad, Saeed Alborzi, Jaleh Zolghadri1, Maryam Parsa-Nezhad, Gholamreza Keshavarzi, Gholamhossein R Omrani, Ernst H Schmidt

**Affiliations:** 1 Division of infertility and GYN endocrinology, Department of Obstetrics and Gynecology, Medical School, Shiraz University of Medical sciences, Shiraz, Iran; 2 Division of cell and Molecular Biology Department of biology, Shiraz University, Shiraz, Iran; 3 Shiraz Health Network, Shiraz, Iran; 4 Endocrine and Metabolism Research Centre, Nemazee Hospital, Medical school, Shiraz, University of Medical sciences, Shiraz, Iran; 5 Division of infertility and GYN endocrinology, Department of Obstetrics and Gynecology, Evang. Diakonie Teaching Hospital of the Göttingen University, Bremen, Germany

## Abstract

**Background:**

The effects of ovarian drilling on the serum levels of gonadotropins and androgens have been studied previously. The aim of this study is to evaluate the effects of ovarian drilling on the serum prolactin levels and its relation to ovulation in women with polycystic ovary syndrome.

**Methods:**

This is a prospective controlled study. Thirty-six women with PCOS underwent ovarian electrocauterization in university hospitals. Control group consisted of 35 ovulatory women with unexplained infertility. Hormonal assessment performed in early follicular phase of spontaneous or induced cycle before operation in the two groups and repeated one week after operation. Hormonal assay was also performed in the early follicular phase of the first post-operative menstruation, folliculometry and progesterone assay were also performed in the same cycle. Data were analyzed by "repeated measurement design, discriminant analysis, correlation coefficient, and Fisher exact test".

**Results:**

Six to ten weeks after operation the serum mean +/- SD prolactin levels increased from 284.41 +/- 114.32 mIU/ml to 354.06 +/- 204.42 mIU/ml (P = 0.011). The same values for the control group were 277.73 +/- 114.65 to 277.4 +/- 111.4 (P = 0.981) respectively. Approximately 45% of subjects in PCOS group remained anovulatory in spite of decreased level of LH and testosterone. Prolactin level remained elevated in 73.2% of women who did not ovulate 6–10 weeks after the procedure.

**Conclusion:**

Hyperprolactinemia after ovarian cauterization may be considered as a possible cause of anovulation in women with polycystic ovaries and improved gonadotropin and androgen levels. The cause of hyperprolactinemia is unknown. Hormonal assay particularly PRL in anovulatory patients after ovarian cauterization is recommended.

## Background

The polycystic ovary syndrome (PCOS) is associated with chronic anovulation and infertility. In most cases ovulation can be induced with clomiphene citrate (CC) but approximately 25% of patients fail to ovulate and require alternative treatment [1]. Human menopausal gonadotropins have been used but the risk of hyperstimulation and multifetal gestation [2]. A variety of surgical options for the treatment of PCOS have been applied during laparoscopy (biopsy, cauterization, laser surgery)[3]. Laparoscopic ovarian drilling (LOD) was first described by Gjonnaess [4]. The reported ovulation rate after LOD varies between 50% and 90% [4-7], the conception rate dose not increase in parallel with the increase in ovulation rate. There is also some disparity between hormonal improvement and ovulation rate [7-9]. Part of disparity may be due to post-operative adhesion formation [10], post-LOD hyperprolactinemia [11], and any unknown reason. Although many studies concerning the endocrine effects of LOD have been performed [12-16], none has emphasized on the cause of disparity between hormonal changes and ovulation. We performed this prospective, controlled study to evaluate the effects of LOD on hormonal profile particularly prolactin and their possible effects on ovulation.

## Materials and methods

This study was performed in the Division of Infertility and Gynecologic Endocrinology, Shiraz University of Medical Sciences, Shiraz Iran. Between January 1998 and November 2003, 102 women with PCOD were recruited but 60 of them were not eligible and excluded. Thus 42 clomiphene-citrate resistant anovulatory women with PCOS were enrolled into this prospective, controlled study. Before laparoscopic ovarian drilling, these women had failed to ovulate with the maximum dose of CC (200 mg/day for 5 days for at least 5 cycles). Polycystic ovary syndrome was diagnosed on the basis of the following criteria: hirsutism, menstrual disturbances (oligo- or amenorrhea), increased plasma circulating androgens, LH/FSH ratio>2.5, and typical ultrasonographic findings [17]. We excluded all women with PRL level >500 MIU/ml.

The control series consisted of thirty-five unexplained infertility that had ovulatory cycles and had been referred for diagnostic laparoscopy. At a minimum, the diagnosis of unexplained infertility implies a normal semenanalysis, objective evidence of ovulation, a normal uterine cavity, bilateral tubal patency, and normal post-coital test. They were chosen because the diagnostic laparoscopy procedure utilized was very similar to the LOD in terms of premedication and anesthesia. Ethic committee for Human Research of the university approved the study and informed consent was taken from each patient. In all patients baseline blood samples were obtained before operation (2–3 days after the commencement of spontaneous or progesterone induced menstrual bleeding) to assess serum levels of LH, FSH, PRL, DHEAS and T. First post-operative blood sample was taken 24 hours after operation. Second sampling was performed one week after LOD, and the third blood sample obtained in the early follicular phase of first post-operative menstrual cycle (approximately 6–10 weeks after operation). If menstruation did not occur till one month after LOD, 100 mg of progesterone would be administered intramuscularly to stimulate menstruation. This cycle was monitored for ovulation using serum progesterone (P) measurement in the mid-luteal phase and folliculometry that was performed on days 14–16 of the first menstrual cycle after operation. The samples were labeled; serum was separated and frozen until the end of study when all of them were assayed by the same kit of radioimmunoassay (RIA). PRL was measured in plasma pool (3 samples separated by 30 minute intervals). Women with PCOS (Group A) were treated with laparoscopic ovarian drilling. Laparoscopic ovarian drilling was performed using two-puncture technique. We used an optic that equipped with operative channel. The laparoscope was introduced through a subumblical incision and a grasping forceps was introduced suprapubically to stabilize the ovary by grasping the ovarian ligament. After assessment of the pelvic structures and tubal patency, an insulated needle connected to a unipolar electrocautery unit was inserted through operative channel of the optic. Eight to ten cautery points 3–4 mm in diameter was created in each ovary with a current of 4 mA applied through the laparoscopic insulated needle. Control group (Group B) underwent diagnostic laparoscopy as standard double puncture method under general anesthesia by the same surgeons and anesthetists.

### Statistical methods

Pre-operative and post-operative clinical and endocrinologic parameters in various sessions were compared using "repeated measurement design (Bonferroni test"). Fisher exact test was used to determine progesterone changes and menstruation in the two groups after procedure. We also used "discriminant analysis" for ovulation as dependent factor and other study parameters. Correlation of prolactin levels with ovulation was compared using "correlation coefficient test".

### Normal values of hormonal levels

FSH = 3–13 mIU/ml, LH = 1.5–12 mIU/ml, Testosterone = 0.2–0.9 ng/ml, DHEAS = 80–350 microgram/dl, Prolactin= 50–450 mIU/ml.

## Results

Of a total of forty-two PCOS women enrolled, 36 completed the study protocol, 6 lost to follow up and were excluded. We also excluded five women in control group who did not complete the measurements. Ultimately, 36 women in PCOS group and 30 women in control group underwent final analysis. Mean age in PCOS and control groups were 24.11 +/- 3.22(range 19 to 31) and 24.5 +/- 3.95(range 19 to 32) respectively. Typical endocrine profiles of baseline, and after operation (24 hours, one week and 6–10 weeks) of the two groups are shown in (Table 1). After operation, PRL levels in the both groups increased significantly at 24 hours (P < 0.001) while the other hormonal profiles showed no such changes. Serum levels of LH (P < 0.001), T (P < 0.001), and also LH/FSH ratio (P < 0.001) decreased to a statistically significant level 6–10 weeks after operation. Comparison of study parameters in PCOS group: women who ovulated spontaneously Vs. women who did not ovulated spontaneously, showed that prolactin levels were significantly higher in anovulatory group and follicle size (F.Size) is grater in ovulatory group (Table 2). Hormonal profiles at baseline and all sessions after operation between PCOS and control group were compared. The results are depicted in Table 3. Twenty-four hours after operation serum PRL levels rose in 88.9% of PCOS and 100% of control group respectively (Table 1). Serum PRL levels remained elevated 6–10 weeks after operation in 27.8% of PCOS (P < 0.05) and 6.7% of control group (P > 0.05)(Table 4). After operation, 20(55.6%) of PCOS and 30 (100%) of control group ovulated as indicated by midluteal serum progesterone level > 5 ng/ml, spontaneous menstruation and leading follicular size >15 mm (Table 4). Of all patients that remained anovulatory in spite of decrease in LH and T after LEC, PRL remained higher than normal limits in 62.5%.

**Table 1:** Hormonal profile(Mean +/-SD) at baseline and various sessions after operation in PCOS and control group (All post operative sessions compared with baseline).

	**0**	**1(P.Value)**	**2(P.Value)**	**3(P.Value)**
**1**-FSH				
PCOS	6.4+/-1.97	6.21+/-1.71(0.904)	6.35+/-1.68(0.616)	6.17+/-1.62(0.812)
Control	6.74+/-1.75	6.81+/-1.39(0.782)	6.58+/-1.79(0.417)	6.47+/-1.24(0.407)
**2**-LH				
PCOS	16.21+/-4.41	14.28+/-4.04(0.001)*	13.95+/-3.54(0.001)*	8.26+/-2.17(0.001)*
Control	8.02+/-1.31	8.32+/-1.68(0.356)	8.31+/-1.98(0.403)	7.78+/-1.49(0.470)
**3**-LH/FSH R				
PCOS	2.71+/-0.58	2.36+/-0.59(0.002)*	2.3+/-0.7(0.001)*	1.42+/-0.47(0.001)*
Control	1.23+/-0.22	1.23+/-0.13(0.961)	1.28+/-0.13(0.295)	1.22+/-0.23(0.897)
**4**-DHEA, S				
PCOS	201.14+/-103.21	199.5+/-98.92(0.772)	195.17+/-9043(0.538)	199.56+/-95.1(0.794)
Control	198.37+/-82.66	186.77+/-73.75(0.262)	187.2+/-79.97(0.157)	191.17+/-68.04(0.306)
**5**-T				
PCOS	1.18+/-0.33	1.12+/-0.31(0.008)*	1.08+/-0.28(0.001)*	0.78+/-0.25(0.001)*
Control	0.66+/-0.23	0.54+/-0.22(0.004)*	0.61+/-0.17(0.095)	0.62+/-0.17(0.081)
**6**-PRL				
PCOS	284.41+/-114.32	651.83+/-316.79(0.001)*	530.31+/-206.74(0.001)*	354.06+/-204.42(0.011)*
Control	277.73+/-114.65	732.23+/-209.91(0.001)*	512.7+/-131.1(0.001)*	277.4+/-111.4(0.981)
**7**-Prog				
PCOS	-	-	-	5.99+/-3.68
Control	-	-	-	8.58+/-2.31

0 = Baseline

1 = Mean+/-SD 24 h after operation

2 = Mean+/-SD one week after operation

3 = Mean+/-SD 6–10 weeks after operation

*= significant P value (P < 0.05)

**Table 2:** Comparison of study parameters in PCOS group. Women who ovulated spontaneously Vs. women who did nor ovulated spontaneously.

	**Anovulatory group**	**Ovulatory group**	**P value**
**1-Age**	24.31 +/- 3.6	23.95 +/-2.92	0.74
**2-FSH**	5.91+/-1.95	6.37 +/-1.31	0.4
**3-LH**	8.06 +/-1.99	8.42 +/-2.33	0.63
**4-DHEAS**	209.125+/-96.033	191.9 +/-96.13	0.59
**5-T**	0.75 +/-0.26	0.8 +/-0.24	0.52
**6-PRL**	501.31 +/-205.130	236.25 +/-104.34	0.000*
**7-F. Size**	9.45 +/-1.48	17.1 +/-1.33	0.000*

**Table 3:** Comparison hormonal profiles(Mean+/-SD) at baseline and various sessions after operation, between PCOS & control group.

	0 (P value)	1(p value)	2 (p value)	3 (p value)
**1**-FSH				
PCOS	6.4+/-1.97 (0.27)	6.21+/-1.71(0.131)	6.35+/-1.68(0.555)	6.17+/-1.62(0.31)
Control	6.74+/-1.75	6.81+/-1.39	6.58+/-1.79	6.47+/-1.24
**2**-LH				
PCOS	16.21 +/-4.41(<0.001)*	14.28+/-4.04(<0.001) *	13.95+/-3.54(<0.001) *	8.26+/-2.17(0.24)
Control	8.02+/-1.31	8.32+/-1.68	8.31+/-1.98	7.78+/-1.49
**3**-DHEAS				
PCOS	201.14 +/-103.2(0.906)	199.5+/-98.92(0.562)	195.17+/-90.43(0.699)	199.56+/-95.1(0.029)
Control	198.37+/-82.66	186.77+/-73.75	187.2+/-79.97	191.17+/-68.04
**4**-T				
PCOS	1.18 +/-0.33(0.001) *	1.12+/-0.31(<0.001) *	1.08+/-0.28(<0.001) *	0.78+/-0.25(0.1)
Control	0.66 +/-0.23	0.54+/-0.22	0.61+/-0.17	0.62+/-0.17
**5**-PRL				
PCOS	284.41+/-114.32(0.814)	651.83+/-316.76(0.239)	530.31+/-206.74(0.688)	354.06+/-204.42(0.006)*
Control	277.73+/-114.65	732.23+/-209.9	512.7+/-131.1	277.4+/-111.4

0 = Baseline 1

= Mean+/-SD 24 h after operation

2 = Mean+/-SD one week after operation

3 = Mean+/-SD 6-10 weeks after operation * = significant P value (P < 0.05)

**Table 4:** Clinical and hormonal outcome 6–10 weeks after operation in the two groups.

	**NOPW L.F>15 mm (%)**	**NOPW Prog>5 ng/ml (%)**	**NOPW S.M (%)**	**NOPW E. PRL (%)**
**1**-PCOS	19(52.8)	20(55.6)*	20(55.6)*	10(27.8)*
**2**-Control	21(70)	30(100)	30(100)	2.(6.7%)

* = significant P Value

Differences compared with pre-operation value

NOPW L.F = Number of Patients With Leading Follicle.

NOPW Prog = Number of Patients With Progesterone

NOWP S.M = Number of Patients With Spontaneous Menstruation.

NOPW E. PRL = Number of Patients With Elevated Prolactin.

## Discussion

This is the first prospective, controlled study on the PRL level after LOD Vs. diagnostic laparoscopy. Our study shows that laparoscopic ovarian drilling can restore ovulation in some but not all PCOS women. This confirms the results from previous studies [4-7]. Kovacs et al [12] in their recent study of comparison of ovulation and pregnancy rate has found no difference in success rates between ovarian drilling and gonadotropin ovulation induction for such women in spite of hormonal improvement after LOD. Abdel Ghadir et al [7] also reported that beside its favorable endocrine effects, ovarian drilling revealed the same rate of ovulation and pregnancy in comparison with HMG induced cycle. In the present study we were able to show that with ovarian drilling, T and LH levels decreased irrespective of ovulation. LH and T decreased in 75% and 70% of PCOS patients respectively, whereas only 52.8% of women who underwent ovarian drilling ovulated. Periovarian adhesion after ovarian electrocautery seems to be the main cause of disparity between ovulation and pregnancy rate. Many authors believe that although periovarian adhesion after ovarian drilling is much less when compared with ovarian wedge resection, still some significant adnexal adhesion that adversely affects fertility may be considered [10,18,19]. The unanswered question concerning the effects of ovarian drilling is the disparity between favorable endocrine effects of this procedure and ovulation rate. Concerning the endocrine effects of LOD, Gjonness et al [20] in their study on 17 women showed a transient hyperprolactinemia immediately after LOD. They believed that this phenomenon was due to operative stress. Eldib et al in their unpublished study on 20 women with PCOS showed that LOD was associated with increased PRL levels by 7 folds two weeks after operation.

We hypothesized that the disparity seen between hormonal improvement and ovulation rate might be due to the hyperprolactinemia associated LOD. Of all operated women that remained anovulatory in spite of significant fall in LH and T levels, PRL remained elevated in 62.5% when measured 6–10 weeks after operation. Serum prolactin level began to rise from the first day after LOD in the two groups. Six to ten weeks after operation, PRL level in the women with PCOS was significantly higher than control group. Hyperprolactinemia as a complication of operation, and/or anesthesia was previously described by Adashi [21], Chan [22], Frantz [23], Newsome [24], Charters [25], and Soules et al [26]. An elevated PRL level was common finding during and after operation with the peak PRL levels always occurring during surgery. The elevated post-operative PRL levels may be explained by a generalized stress reaction (e.g., pain) and by medications known to stimulate PRL release (e.g., narcotics) [27]. We did not measure PRL intra-operatively, but post-operative elevated levels of PRL support the results of previous studies. Noel et al [28] and Sowers [29] reported an acute increase in PRL concentration with the induction of anesthesia prior to the operative incision. The operative increase in PRL was greater in women than in men and persisted for a various periods [28]. The post-operative elevated PRL levels improved spontaneously after a maximum period of one week. Approximately all subjects in the two groups and the most cases of PCOS had normal PRL 6–10 weeks after LOD. A large number of women with PCOS, who remained anovulatory after LOD, had still elevated serum PRL levels. Gjonnaess in his study of long term follow up showed no difference between baseline PRL levels and PRL levels after 3 months or later after LOD [30]. The disparity between this study and our results may be due to the longer duration between LOD and PRL assay in Gjonnaess group. Since the LOD and control group were in the same condition concerning the surgical and anesthesiologic stress, and the post-operation elevated PRL level was supposed to decrease after one week, the elevated PRL levels 6–10 weeks after operation in LOD group may be due to other cause than surgical or anesthesiologic stress. The mechanism for the increase in PRL levels after LOD remains speculative. The possible mechanism might be scar formation on the surface of ovaries and chronic stimulation of ovarian nerves and causing neurogenic hyperprolactinemia, like chest wall lesion, intercourse or spinal cord lesions. For documentation of this hypothesis animal models should be designed.

In this prospective controlled study control group helped us to differentiate stress-induced hyperprolactinemia from the hyperprolactinemia associated with LOD. The weak point of this preliminary report is that the hormonal assay was performed in early and late post-operative phase. More exact hormonal study is necessary to evaluate the condition. However this study provides a potential new way to evaluate the factors that affecting ovulation rate after LOD including hyperprolactinemia. It seems that animal study to evaluate the relationship between ovarian damage and PRL levels would be helpful. Increase in serum PRL levels associated with LOD has several implications in clinical practice; 1- post-operative elevated PRL levels affects gonadotropins and ovarian steroidogenesis [31,32], so it would be prudent not to rely on early hormonal profiles after LOD, 2- Since the latent hyperprolactinemia may lead to luteal phase defect [33] if the first cycle after LOD was ovulatory, luteal phase defect must be considered and treated. 3- If ovulation did not occurred as expected, diagnosis and treatment of hyperprolactinemia should be undertaken.

## Conclusion

We conclude that after ovarian drilling, women who remained anovulatory in spite of decreased serum androgen levels and other hormonal profile improvement, may have elevated prolactin levels. The cause of hyperprolactinemia in these patients has not known. Hormonal assay particularly PRL in anovulatory patients after LOD is recommended. Animal study to evaluate the relationship between ovarian damage and PRL levels would be helpful

## Authors' contributions

M.E.Parsanezhad,. Conception and design, Lapascopies.

S. Alborzi, Conception and design of the study and referring patients to our clinic.

Jaleh Zolghadri. Conception and design, coordination and helped to draft the manuscript.

Maryam Parsa-Nezhad. Carried out the hormonal assays, participated in the sequence alignment.

Gh.Keshavarzi. Carried out the hormonal assays.

GH R.Omrani. Carried out the hormonal assays.

E.H.Schmidt. He has been involved in revising the manuscript.
